# The combination of modified acupuncture needle and melittin hydrogel as a novel therapeutic approach for rheumatoid arthritis treatment

**DOI:** 10.1186/s12951-024-02722-y

**Published:** 2024-07-22

**Authors:** Lisha Liu, Dashi Deng, Chenchen Li, Guixiao Huang, Wenjuan Zhang, Ting Liang, Rui Liang, Mingkang Liang, Yilin Su, Chongyang Lin, Guangzhi Li, Song Wu

**Affiliations:** 1 Institute of Urology, The Affiliated Luohu Hospital of Shenzhen University, Shenzhen University, Shenzhen, 518000 China; 2 Institute of Urology, South China Hospital, Health Science Center, Shenzhen University, Shenzhen, 518116 China

**Keywords:** Acupuncture, Rheumatoid arthritis, Melittin, Drug delivery, Hydrogel

## Abstract

Rheumatoid arthritis (RA) involves chronic joint inflammation. Combining acupuncture and medication for RA treatment faces challenges like spatiotemporal variability, limited drug loading in acupuncture needles, and premature or untargeted drug release. Here, we designed a new type of tubular acupuncture needles, with an etched hollow honeycomb-like structure to enable the high loading of therapeutics, integrating the traditional acupuncture and drug repository into an all-in-one therapeutic platform. In these proof-of-concept experiments, we fabricated injectable hollow honeycomb electroacupuncture needles (HC-EA) loaded with melittin hydrogel (MLT-Gel), enabling the combination treatment of acupuncture stimulation and melittin therapy in a spatiotemporally synchronous manner. Since the RA microenvironment is mildly acidic, the acid-responsive chitosan (CS)/sodium beta-glycerophosphate (β-GP)/ hyaluronic acid (HA) composited hydrogel (CS/GP/HA) was utilized to perform acupuncture stimulation and achieve the targeted release of injected therapeutics into the specific lesion site. Testing our therapeutic platform involved a mouse model of RA and bioinformatics analysis. MLT-Gel@HC-EA treatment restored Th17/Treg-mediated immunity balance, reduced inflammatory factor release (TNF-α, IL-6, IL-1β), and alleviated inflammation at the lesion site. This novel combination of modified acupuncture needle and medication, specifically melittin hydrogel, holds promise as a therapeutic strategy for RA treatment.

**Graphical abstract:**

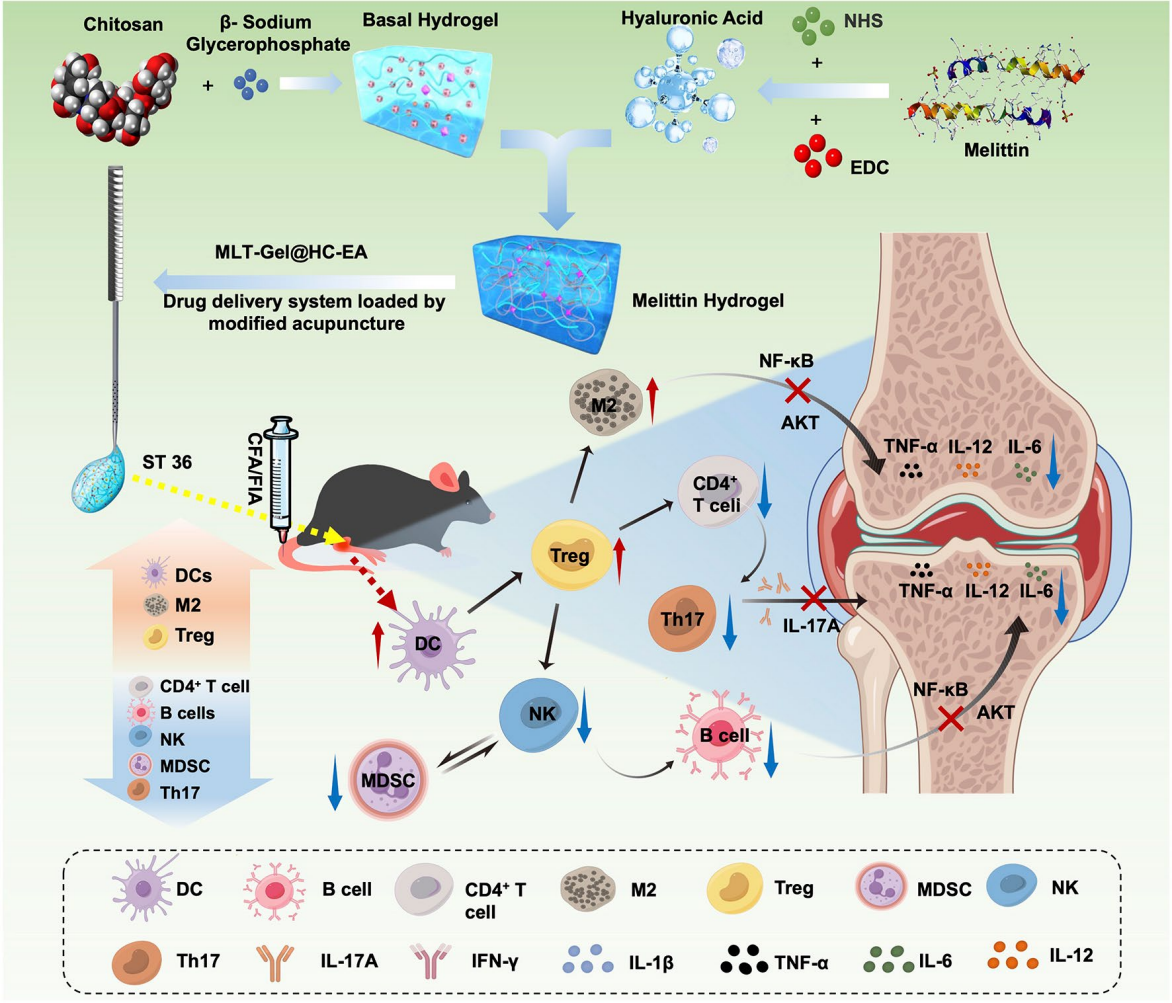

**Supplementary Information:**

The online version contains supplementary material available at 10.1186/s12951-024-02722-y.

## Introduction

Rheumatoid Arthritis (RA) is a chronic autoimmune disease characterized by joint pain, swelling, and deterioration, which impact the quality of life in approximately 1% of global population, resulting in substantial health care burdens worldwide [1]. The management of RA in clinical practice commonly involves the use of glucocorticoids, non-steroidal anti-inflammatory drugs, and biologics. However, these treatments only partially relieve severe symptoms, and the issues related to side effects and frequent relapses have not been solved [2, 3]. Acupuncture has gained widespread acceptance as a treatment option for several non-infectious chronic diseases and, as a result, is currently attracting increasing attention in the area of complementary and alternative medicine research [4]. The classical acupuncture needle (CA-needle) is typically made from solid stainless steel and ranges in length from a few millimeters to several hundred millimeters. To treat numerous health issues, these needles are inserted through the skin surface to reach deep-seated muscles to stimulate acupoints [5]. Acupuncture has demonstrated effective therapeutic benefits, particularly in RA, with no apparent adverse effects [6, 7]. Recently, the combination of acupuncture and drug therapy has gained considerable attention as a result of traditional Chinese and Western medicinal treatment integration.

The latest research has established that the combined use of acupuncture and drug therapy provides significant therapeutic benefits for conditions, such as insomnia, pain, nerve disorders, and osteoarthritis (OA) [8–11]. These approaches are usually restricted by, for example, the time delay between acupuncture stimulation and drug therapy, non-targeted drug distribution in healthy regions, and potential negative drug reactions. Possible improvements to this combined acupuncture-drug approach include creating an anodized layer with multiple micro/nano pores on the surface of the CA-needle [12], injecting triptolide nanogels into acupoints [13], depositing lidocaine on the CA-needle surface using electrochemical deposition [14], and modifying the CA-needle with helical grooves for internally attaching light-cured hydrogels [15]. Despite a recent progress in integrative combination of acupuncture and drug treatment, there are still several limitations, such as drug loading capacity and premature drug release.

**Fig. 1:**
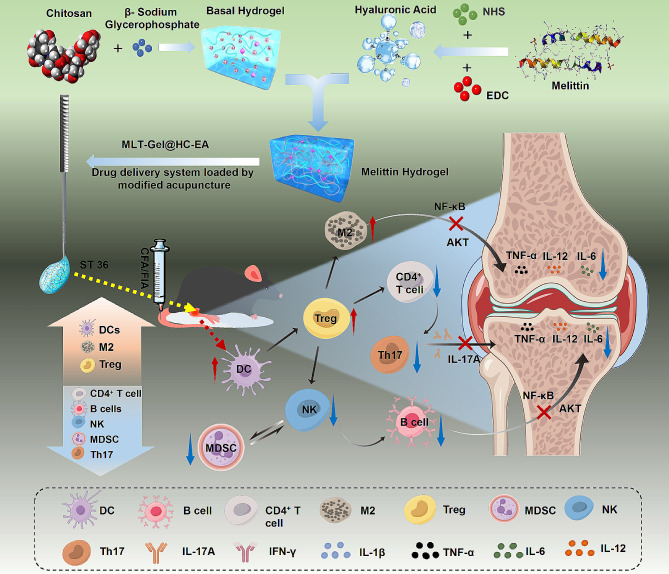
Preparation and mechanism of MLT-Gel@HC-EA for treating RA by Figdraw (www.figdraw.com)

Here, we used a honeycomb design to develop a novel type of hollow injectable acupuncture needle to improve its drug-carrying capacity. To address the acidic RA microenvironment, we designed a pH-responsive hydrogel using chitosan (CS), β-glycerophosphate (β-GP), and hyaluronic acid (HA). As a model drug for the combination system, we selected melittin (MLT), the main component of bee venom and a well-known natural remedy for RA. MLT, mixed with CS/GP/HA-Gel to form MLT-Gel, was loaded onto the honeycomb needle (HC-needle), generating the MLT-Gel@HC-electroacupuncture (EA) delivery system (Fig. 1). This approach combined EA with MLT-gel, allowing both therapies to act simultaneously in a synergistic manner. Furthermore, the acidic immune microenvironment of RA facilitated passive MLT release, leading to the targeted release at the lesion site. This design of the MLT-gel not only extended the duration of acupuncture stimulation through volume effects, but also facilitated slow drug release. Finally, the ability of the HC needles significantly enhanced its drug-carrying capacity, allowing for repeated, high-dose drug load. The effectiveness of MLT-Gel@HC-EA was confirmed using animal experiments, which demonstrated promising results compared to either single EA or MLT injection. MLT-Gel@HC-EA treatment successfully suppressed the activation of natural killer (NK) cells, diminished the function of Th17 cells, enhanced dendritic cell (DC)-induced T-cell immune tolerance, stimulated the expansion of Regulatory T (Treg) cells, and, hence, restored the Th17/Treg ratio. Moreover, it boosted polarization of M2 macrophages, suppressed the secretion of inflammatory factors Tumor Necrosis Factor-α (TNF-α), Interleukin-6 (IL-6), and Interleukin-β (IL-1β), and regulated immune cells, potentially by inhibiting AKT and NF-κB signaling pathways. Our investigation offers valuable insights into the integration of traditional Chinese and contemporary medicine, presenting an inventive therapeutic approach for controlling the RA immune microenvironment.

## Methods

### Materials

Conventional experimental reagents and consumables were obtained from Sigma Aldrich Co. (St. Louis, MO, USA). The special reagents used in the experiments are listed below. HA, NHS, and EDC were obtained from Macklin Co., Ltd (Shanghai, China). Chitosan and β-glycerophosphate were purchased from Aladdin Co., Ltd (Shanghai, China). Melittin (MLT, > 95%) was purchased from Qyaobio Co., Ltd (Shanghai, China), CA-needles were obtained from Yuwell Co., Ltd (Shanghai, China). All C57BL/6J mice used in the study were purchased from the Animal Experimentation Center (Shenzhen, China) and housed under pathogen-free conditions. All animal experimental procedures were approved by the Animal Experimentation Committee of the Third Affiliated Hospital of Shenzhen University.

### Preparation of HC-needles

HC-needles were produced by Yinli Metal, Co., Ltd (Shenzhen, China), a metal processing factory, and 314 medical-grade stainless steel was identified following several trials as the optimal material. The HC-needles had a length of 50 mm and a diameter of 0.6 mm, featuring four rows of pores, with each row consisting of four 0.1-mm diameter pores. These parameters were used as reference points and could be adjusted depending on specific needs.

### Preparation of composite hydrogel

To prepare the hydrogel, 0.1 g of CS was dissolved in 4.5 mL of 0.1 mol/L hydrochloric acid, followed by the addition of 1.5 mL of 56% (w/v) β-GP solution at a slow rate in a dropwise manner while stirring at 240 rpm. Next, 0.1 g of HA powder was dissolved by stirring in 5 mL of deionized water, and then added to the solution. EDC/NHS (mass ratio 2:1) and MLT (45 µg) were dissolved in 1 mL of water, and both EDC/NHS and MLT were added to the HA solution in a dropwise manner (the molar ratio of EDC to NHS monomer was 10: 1). The HA/MLT/EDC/NHS solution was slowly added to the stirred CS/GP solution at a 1:1 ratio. Finally, the free-flowing injectable homogeneous hydrogel CS/GP/HA/MLT-Gel was obtained after approximately stirring for 2 min. These procedures were performed at 4 °C using an ice bath. To solidify the gel, the CS/GP/HA/MLT-Gel was submerged in a water bath at 37 °C for 20 min.

### Characterization of hydrogels

First, the CS/GP/HA/MLT-Gel samples were incubated in a 37 °C water bath for 20 min to allow the formation of a solid gel. Next, the samples were frozen at -20 °C for 24 h, followed by dehydration using a freeze-dryer (SJIA-10–59 A, Shuangjia Instruments Co., Ltd). Samples were coated with a thin layer of gold as previously described [16], and the microstructure of the gel surface was observed using SEM (JEOL 7600 F with Gatan Alto).

### In vitro release profile

To simulate the release of MLT from CS/GP/HA/MLT-Gel in vivo, we conducted MLT in vitro release experiments using the release media and various pH parameters, and measured the concentration of released MLT. First, 5 mL of CS/GP/MLT-Gel and CS/GP/HA/MLT-Gel were placed into a solution (5 mL) with pH 7.4 (physiological) and pH 5.5 (inflammatory). Prepare a pH 5.5 solution by diluting hydrochloric acid (HCl) with distilled water and ilute 10× PBS with distilled water to prepare a neutral solution with a pH of 7.4. Hydrogel-containing glassware was incubated at 37 °C, and MLT concentration in the solution was measured at different time points using UV/Visible Photometer-5100 (METASH, China). The cumulative release of MLT was calculated and all the experiments were repeated three times.

### Hydrogel swelling

Begin by measuring the tare weight of a 20-milliliter glass vial. Following the previously outlined in vitro release experimental procedure, prepare solutions with pH values of 7.4 and 5.5. Into the glass vial, introduce 5 milliliters of hydrogel, allowing it to solidify at 37 °C for 20 min. Following solidification, add 5 milliliters of the prepared solution. The glass vial containing the loaded hydrogel was then positioned in a controlled temperature environment set to 37 °C. At predetermined intervals, the suspension was extracted, excess water was blotted using a filter paper, and the hydrogel was weighed. Next, to determine the net weight loss, the hydrogel was dried using a freeze dryer. The dried samples were then resuspended in pH 7.4 and 5.5 solutions and incubated at 37 °C for 24 h. The samples were subsequently removed, dried to remove excess moisture, and weighed. The weighing process was repeated until a constant weight was achieved.

#### In vivo biodistribution of MLT-gel

To label CS/GP/HA/MLT-Gel, 50 µg of Cy-5.5 was dissolved in 1 mL of PBS solution (pH 7.4) containing 1 mg of MLT and stirred in the dark at 25 °C for 12 h. The excess Cy-5.5 dye was removed using a dialysis membrane (MWCO = 1000 Da). Next, the solution was added in a dropwise manner to the prepared CS/GP/HA-Gel. Mice were injected with MLT solution or MLT-Gel (40 µl each) either at the acupoint (ST36) or non-acupoint (subcutaneous region near the tail base). Live-animal imaging was conducted at 2, 8, 12, 24, 48, 72, and 96 h using the in vivo imaging system (AniView100, BioLight Instruments Co., Ltd). To evaluate MLT distribution in mouse organs, mice were euthanized at 2, 8, 24, and 48 h, organs, such as the heart, liver, spleen, lungs, kidneys and paws, were collected, and fluorescence signals were observed using the in vivo imaging system.

### Hydrogel transport test

A 15 × 15 × 10 mm piece of muscle tissue was dissected from the hind limb of a domestic pig. Puncture experiments were conducted at two sites using CA and HC needles to simulate acupuncture in clinical practice. Both CA and HC needles were loaded with CS/GP/MLT-Gel labelled with the Cy-5.5 fluorescent dye. The vivo imaging system (AniView100, BioLight Instruments Co., Ltd) was used to detect and record the initial fluorescence intensity at the tips of both needles. Subsequently, the fluorescence intensity of both microneedles and porcine tissues were measured before and after needle insertion, and the fluorescence intensity of the gel delivered to the interior of the porcine tissue was calculated.

### Mouse RA model

Dissolve chicken type II collagen (Biolead, China; 20,012) in acetic acid (2 mg/mL) and stir thoroughly at 4 °C. After complete dissolution, refrigerate overnight at 4 °C. Mix the collagen solution with an equal volume of CFA and perform ultrasonic emulsification. On day 0, inject the emulsion subcutaneously at the base of the mouse tail with a dosage of 0.1 ml per mouse for the first immunization. On the 7th day of the experiment, inject 0.1 ml of chicken type II collagen mixed with Incomplete Freund’s Adjuvant (IFA) per mouse into the footpads for booster immunization. The mixing and emulsification methods are the same as those used in the first immunization. Mice in the normal control group are injected with PBS solution in the same manner. Monitor the progression of arthritis in mice daily, ensuring that all mice have sufficient food and water during disease development. The successful modeling of RA is indicated by the appearance of joint redness and swelling in mice.

### Mouse acupuncture experiment

**Fig. 2:**
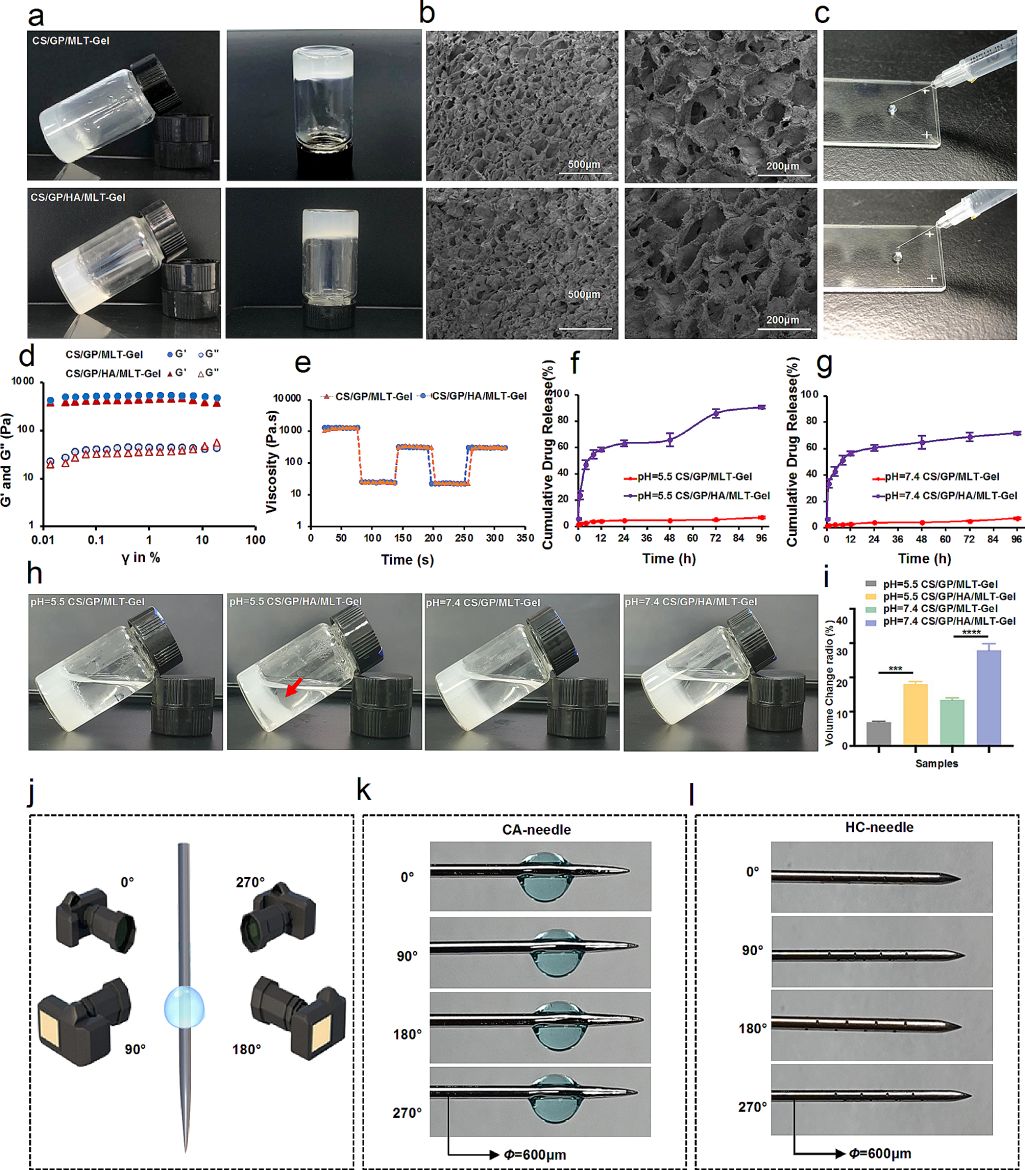
fabrication and characterization of composite hydrogels. (**a**) representative macroscopic images of cs/gp/mlt-gel and cs/gp/ha/mlt-gel. (**b**) representative sem images of cs/gp/mlt-gel and cs/gp/ha/mlt-gel. (**c**) representative images of cs/gp/mlt-gel and cs/gp/ha/mlt-gel passed through a 25 g syringe needle. (**d**) shear rheology results of cs/gp/mlt-gel and cs/gp/ha/mlt-gel analysis. (**e**) step-shear results of cs/gp/mlt-gel and cs/gp/ha/mlt-gel analysis. (**f**) release profile of mlt in acidic environment. (**g**) release profile of mlt in neutral environment. (**h**) representative macroscopic images of cs/gp/mlt-gel and cs/gp/ha/mlt-gel at ph 5.5 and 7.4. (**i**) swelling coefficient of cs/gp/mlt-gel and cs/gp/ha/mlt-gel at ph 5.5 and 7.4. (**j**) a diagram depicting the angles used to acquire 360° images of the acupuncture needle. (**k**) the cs/gp/ha/mlt-gel was placed onto the ca-needle and images were obtained as described in (j). (**l**) the cs/gp/ha/mlt-gel was placed onto the hc-needle and images were obtained as described in (j)

Mice were divided into the following seven groups (*n* = 6/group): the Control group (ST36 PBS injection), the RA PBS group (ST36 PBS injection), the RA EA group (ST36 electroacupuncture), the RA Blank-Gel@HC-EA group (ST36 electroacupuncture with Blank-gel), the RA MLT group (tail MLT solution injection), the RA MLT-Gel group (ST36 MLT-Gel injection), and the MLT-Gel@HC-EA group (ST36 electroacupuncture with MLT-Gel). The ST36 acupoint is located between the tibia and fibula of the hind limb of the mouse, approximately 3 mm from the ankle joint (Fig. 2). As previously described, the mice were subjected to anesthesia with inhaled isoflurane (0.5–1.5%), while a heating pad was utilized to maintain their body temperature [17]. Following acupuncture needle insertion, the electroacupuncture instrument (SDZ-II, Suzhou Medical Supplies Co., Ltd) was connected, and parameters were set to 2 mA current intensity, 2 Hz frequency, sparse and dense waveforms, and a 30-minute treatment time. Mice were treated every 4 days, for a total of four treatments.

### Histological staining analysis

On day 28, mice were euthanized by CO_2_ and major organs, including the heart, liver, kidneys, lungs, and spleen, were harvested and fixed in 4% paraformaldehyde for subsequent H&E staining. Bilateral ankle joints were fixed in 4% paraformaldehyde for 48 h and decalcified in a 10% neutral EDTA solution at room temperature for 15 days. The decalcified tissues were then embedded in paraffin, sectioned and then stained with H&E, SO-FG, Masson, and T&B according to the manufacturer’s protocol. Sections were examined under a microscope (BX43, OLYMPUS Co., Ltd). Synovial inflammation, bone erosion, and cartilage degradation were evaluated and tissue pathology scores were assigned (Table S1).

### ELISA analysis

For the mice in the in vivo biodistribution experiment, orbital blood was collected before administration and at the point of sacrifice. For the mice in the therapy experiment, orbital blood was collected at the following time points: before modeling (0 days), after modeling (7 days), and after treatment (28 days). The samples were centrifuged at 1,000 rpm at 4 °C for 5 min, and 200 µL of serum was collected. The following primary antibodies were used: BUN (CDEbio, China; EN-XS91949), CR (CDEbio, China; EN-XS91786), ALT (CDEbio, China; EN-XS91621), AST (CDEbio, China; EN-XS91621), SP-A (CDEbio, China; EN-XS91701), SP-D (CDEbio, China; EN-XS91703). CCP-Ab (CDEbio, China; EN-XS92425), CRP (CDEbio, China; EN-XS91470), RF (CDEbio, China; EN-XS91889), ANA (CDEbio, China; EN-XS91853), TNF-α (Mlbio, China; mIC50536), IL-12 (Mlbio, China; ml037868), IL-17 A (Mlbio, China; ml037864), IL-1β (Mlbio, China; mIC50300-1), and IFN-γ (Mlbio, China; ml064291).

### Immunohistochemical analysis

The specimens were deparaffinized and antigen retrieval was performed using a microwave. Next, the sections were blocked with 3% BSA for 30 min and then incubated with primary antibodies against TNF-α (Servicebio, China; GB11188), IL-Iβ (Servicebio, China; GB1113), and IL-6 (Servicebio, China; GB11117) overnight at 4 °C. The sections were washed, the biotinylated secondary antibody was added, and samples were incubated for 50 min. Samples were then incubated with the streptavidin solution. 3, 3-diaminobenzidine tetrahydrochloride (DAB, 10 µL) was used as a chromogen and samples were counterstained with hematoxylin. The sections were dehydrated using alcohol gradients and then mounted. The slides were visualized under the microscope (BX43, OLYMPUS Co., Ltd), and images of three randomly selected fields of view for each sample were obtained. The staining intensity was determined using the ImageJ software (National Institutes of Health, USA).

### Cell flow analysis

Spleens were collected from mice, processed, and filtered to prepare a single-cell suspension. Erythrocytes were lysed and the cells were incubated with a fluorescent conjugated antibody at 4 °C in the dark for 60 min. Next, the cells were washed with cold PBS and cells were analyzed using the BD Canto II flow cytometer. The precise antibodies utilized in this experiment were FITC-anti-CD19 (Biolegend, USA; 101,505), Percp-anti-CD45 (Biolegend, USA; 103,130), PE-anti-CD45R (Biolegend, USA; 103,207), PerCP/Cyanine5.5 anti-CD3 (Biolegend, USA; 100,217), FITC-anti-CD3 (Biolegend, USA; 100,203), PE-anti-CD4 (Proteintech; PE-65,104), APC anti-CD8 (Proteintech, USA; APC-65,069), APC-anti-CD25 (Biolegend, USA; 162,105), Alexa Fluor^®^488 Anti-Foxp3 (Biolegend, USA; 126,405), Apc- anti-IL-17 A (Biolegend, USA; 506,916), FITC-anti- CD11c (Biolegend, USA; 117,305), APC-anti-MCHII (Invitrogen, USA; 17-53281-81), PerCP anti-CD3ε (Biolegend, USA; 100,325), FITC-anti- NK1.1 (Biolegend, USA; 108,705), APC-anti-F4/80 (Invitrogen, USA; 17-4801-82), PE-anti-CD207 (Invitrogen, USA; 12-2075-82), PE-anti-CD45 (Proteintech; PE-65,087), Percp-anti-CD11b (Biolegend, USA; 101,230), FITC-anti-Ly6Gr1 (Proteintech; FITC- 65,140).

### Bioinformatics analysis

RA-related target genes were collected from UniProt (https://www.uniprot.org/), DisGeNET (https://www.disgenet.org/), CTD (https://ctdbase.org/), and GeneCards (https://www.genecards.org/) databases. MLT-related target genes were obtained from the BATMAN-TCM data (http://bionet.ncpsb.org.cn/batman-tcm/). Venny 2.1.0 (https://bioinfogp.cnb.csic.es/tools/venny/) tool was used to identify overlapping genes between constituent targets and disease targets. A protein-protein interaction (PPI) network was constructed using the STRING database (https://string-db.org/). Cytoscape 3.7.1 (Cytoscape Consortium, USA) was utilized to visualize and analyze the interconnection network data exported from the STRING database. The overlapping genes were uploaded onto the DAVID (https://david.ncifcrf.gov/home.jsp, version 6.8) database to conduct a gene ontology (GO) enrichment analysis. In addition, the same genes were also uploaded onto the Kyoto Encyclopedia of Genes and Genomes (KEGG, https://www.kegg.jp/) database for enrichment analysis [18]. 

### Molecular docking

The 3D structures of the core target genes and associated MLT proteins were obtained from the PDB database (https://www.rcsb.org). PyMol (DeLano Scientific LLC, USA) was utilized to process the files by eliminating hetero and water molecules, performing hydrogenation, and saving them in the “pdb” format. We also used the ZDOCK server (http://zdock.umassmed.edu/) to facilitate the molecular docking of the ligand and receptor and calculate the binding energy. Subsequently, a reduced binding energy indicated that the docking had been rendered more stable. If the binding energy was less than 0 kcal/mol (1 cal = 4.2 J), it indicated spontaneous binding between the ligand and receptor. A binding energy lower than − 5 kcal/mol suggested good binding activity, and if it was less than − 7 kcal/mol, it indicated strong binding activity [19]. Partial molecular docking results were visualized using PyMol.

### Real-time fluorescent ouantitative PCR (qPCR)

Total RNA was extracted from mouse ankle joint tissues using TRIzol reagent (Invitrogen), with the concentration and OD value (A260/A280) measured using an ultramicro nucleic acid protein detector, targeting a ratio between 1.8 and 2.0. The RNA concentration was adjusted to 300 ng/µL. Reverse transcription was performed using the RevertAid First Strand cDNA Synthesis Kit (Thermo Fisher), and the cDNA was stored at − 20 °C.The qPCR reactions were conducted in a total volume of 20 µL, consisting of 10 µL 2×TransStart Top Green qPCR Super Mix (TransGen Biotech), 0.5 µL each of upstream and downstream primers, 8 µL ddH_2_O, and 1 µL cDNA. Data analysis was performed using the 2^−ΔΔCt^ method to determine the relative expression levels of the top ten targets identified in bioinformatics analysis. Primer sequences are listed in Table S3.

### Immunofluorescence analysis

Tissue samples were fixed in formalin, embedded in paraffin, and then cut into 5 μm sections. Tissue sections were deparaffinized, rehydrated, washed with PBS, and then blocked with 1% normal goat serum in 10% PBS at room temperature for 1 h. Next, primary antibodies were added and samples were incubated overnight at 4 °C. The samples were washed three times with PBS (10 min/wash) and then incubated with secondary antibodies conjugated to Alexa-488 or Alexa-594 at room temperature for 1 h. The sections were washed, mounted using DAPI-containing anti-fade mounting solution, and then imaged using a fluorescence microscope (Nikon Eclipse Ti-SR, Japan). The following primary antibodies were used: anti-NF-κB (Servicebio, China; GB11997), anti-IL-Iβ (Servicebio, China; GB1113), and anti-AKT (Servicebio, China; GB15689).

### Statistical analysis

Each group included at least three independent samples. To verify normal distribution, we conducted column statistics on the data sets. All data are presented as the mean ± standard deviation (SD) and analyzed using one-way ANOVA with the Bonferroni test employed for multiple comparisons. Statistical analyses were performed using Excel (Microsoft, USA), SPSS (IBM Corp, USA), and GraphPad Prism 9 (GraphPad Software, USA). Statistically significant differences were considered at *p < 0.05.

## Results

### Preparation and characterization of MLT-Gel@HC-EA

Using a previously published protocol, we prepared a thermosensitive CS/GP-Gel hydrogel by mixing CS and β-GP [20]. Next, the carboxyl groups of HA were activated using the crosslinking agents N-(3-Dimethylaminopropyl)-N′-ethylcarbodiimide hydrochloride (EDC)/N-Hydroxysuccinimide (NHS), and HA was incorporated into the CS/GP-Gel, resulting in a composite hydrogel (CS/GP/HA-Gel) [21]. Finally, an MLT aqueous solution was introduced separately into either CS/GP or CS/GP/HA hydrogels to form CS/GP/MLT-Gel and CS/GP/HA/MLT-Gel, respectively (Fig. 2-a). Scanning electron microscopy (SEM) showed that both CS/GP/MLT-Gel and CS/GP/HA/MLT-Gel had a porous and irregular lattice-like structure (Fig. 2-b). At 25°C, both hydrogels had a fluid-like consistency and could pass through a 25G syringe needle without an obstruction (Fig. 2-c). The results of strain-dependent oscillatory shear rheology demonstrated that, as the frequency of oscillation increased, both CS/GP/MLT-Gel and CS/GP/HA/MLT-Gel had a higher storage modulus (G′) than loss modulus (G′′), presenting the elastic mechanical properties of a solid under oscillatory conditions. The CS/GP/MLT-Gel exhibits a consistently large difference between G’ and G’’, indicating high structural stability and low viscous loss. The CS/GP/HA/MLT-Gel demonstrates solid-like characteristics at low frequencies, while exhibiting appropriate viscosity at high frequencies, facilitating effective drug release (Fig. 2-d). Step-shear measurements showed that both CS/GP/MLT-Gel and CS/GP/HA/MLT-Gel rapidly regained their initial viscosity at low shear rates, indicating their excellent self-healing properties (Fig. 2-e).

Next, the drug-release properties of CS/GP/MLT-Gel and CS/GP/HA/MLT-Gel were evaluated in vitro. Under pH 5.5 conditions, the drug release from CS/GP/HA/MLT-Gel was significantly higher than from CS/GP/MLT-Gel (Fig. 2-f). Under pH 7.4 conditions, the drug release from CS/GP/HA/MLT-Gel was also higher than from CS/GP/MLT-Gel (Fig. 2-g), but the overall release was lower compared to that at pH 5.5, indicating that CS/GP/HA/MLT-Gel has a lower responsiveness under neutral conditions and potentially better responsiveness to acidic environments. The integrity of CS/GP/HA/MLT-Gel was visibly affected in response to acidic conditions (pH = 5.5), leading to its disintegration and dissolution; however, the CS/GP/MLT-Gel maintained its integrity in both acidic and neutral conditions (Fig. 2-h). Further experiments showed that the swelling coefficient of CS/GP/HA/MLT-Gel was significantly higher than that of CS/GP/MLT-Gel (Fig. 2-i). The CS/GP/HA/MLT-Gel demonstrated a significantly better drug release and solubility, both in acidic and neutral environment, compared to that of the CS/GP/MLT-Gel. Since the release levels of CS/GP/MLT-Gel were very low in both acidic and neutral environments, it is suggested that it does not exhibit pH responsiveness. To verify whether CS/GP/HA/MLT-Gel is pH-responsive, further in vivo experiments are needed.

To fabricate MLT-Gel@HC-EA, the acupuncture needle-drug delivery system, a drop of liquid CS/GP/HA/MLT-Gel was placed onto the HC-needle using a 1-mL syringe (Fig. 2-j). The hydrogel loaded onto the CA-needle was visible on the metal surface, protruding beyond the contour of the CA-needle (Fig. 2-k); however, the hydrogel loaded onto the hollow HC-needle did not noticeably extend beyond the contour of the HC-needle (Fig. 2-l). These findings suggested that the loaded hydrogel could be protected from premature leakage during tissue puncture by the honeycomb porous structure of the HC-needle.

### In vivo biodistribution of MLT-gel and drug delivery performance of HC-needles

**Fig. 3:**
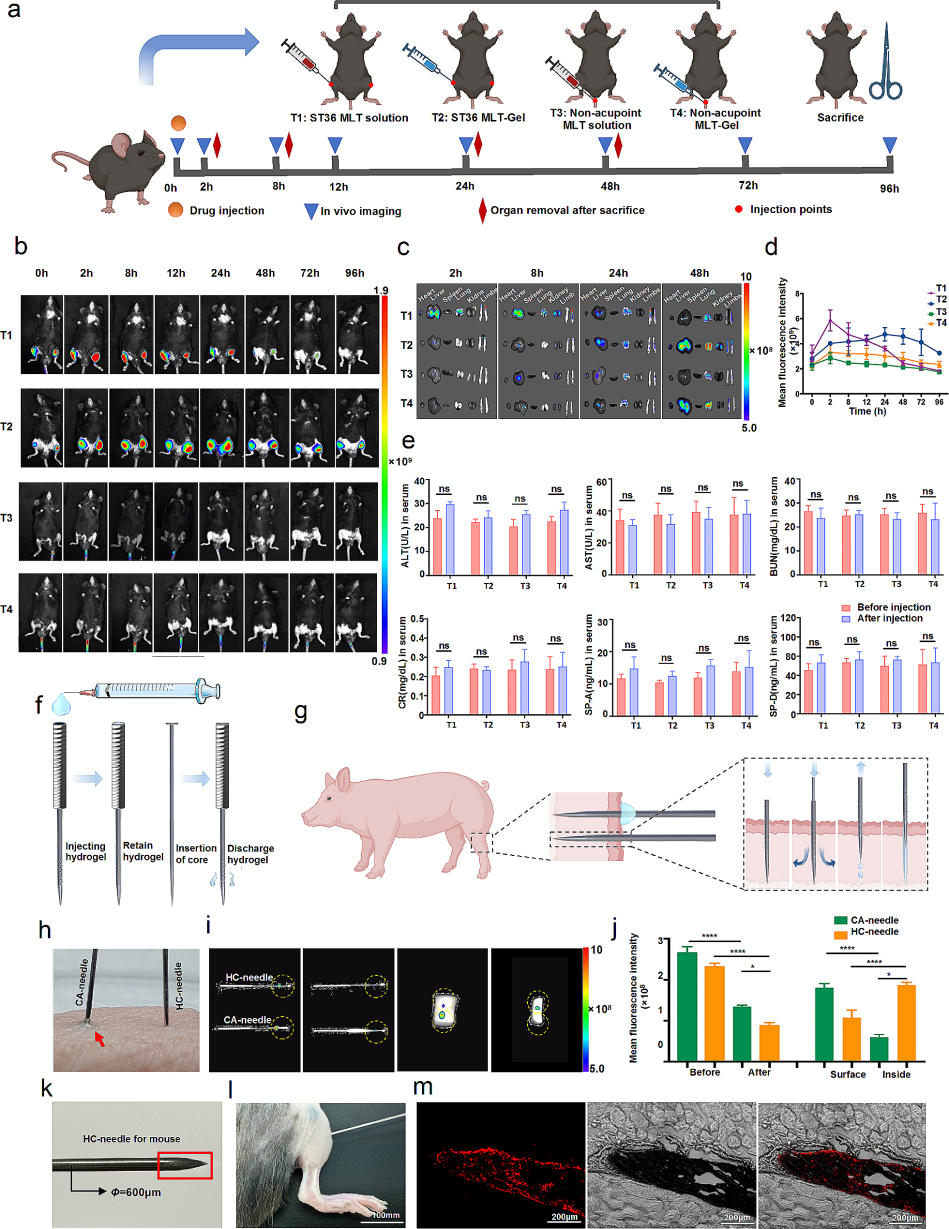
biodistribution of mlt and characterization of hc-needle. (**a**) schematic representation of the mouse biodistribution experiment. (**b**) imaging of ra mice post-administration. (**c**) mice were injected as described in (b) and major organs were collected as the specified time points; the fluorescence intensity of the organs was evaluated using the in vivo imaging system. representative images are shown. (**d**) quantification of fluorescence intensity of the in vitro mlt release experiments. (**e**) analysis of serum safety indicators (alt, ast, bun, cr, sp-a, and sp-d) before and after administration. (**f**) appearance and schematic diagram of hc-needle. (**g**) schematic diagram of the hc-needle transport hydrogel. (**h**) the image depicting the mlt-gel remaining on the skin surface after ca- and hc-needle insertion into the muscle (the gel remaining after ca-needle insertion is indicated by the red arrow). (**i**) cy-5.5-labeled mlt-gel-loaded ca- and ha-needles were used to pierce the porcine tissue sample and the fluorescent signal was imaged; representative images are shown. (**j**) quantification of the fluorescence intensity of the experiments described in (**i**). (**k**) hc-needle for mouse (red-marked areas indicate the locations of retained needle holes). (**l**) macroscopic image of hc-needle insertion at mouse st36. (**m**) fluorescent image of mlt-gel retained at the local acupoint

Since RA primarily affects the joints, the acupoint *Zusanli* (ST36), located below the knee, could be used as an important drug delivery point for the treatment of RA. First, we investigated the effect of drug administration location (acupoint versus non-acupoint) on MLT release dynamics. The RA mouse model was generated as previously described [22]. 16 RA model mice were randomly divided into 4 groups (*n* = 4/group): the T1 group (ST36 MLT solution injection), the T2 group (ST36 MLT-Gel injection), the T3 group (non-acupoint MLT solution injection), the T4 group (non-acupoint MLT-Gel injection). Using an in vivo imaging system, we evaluated the spatial distribution of MLT labeled with the fluorescent dye Cy-5.5 after the administration of MLT solution or MLT-Gel (40 µl each) either at the acupoint (ST36) or non-acupoint (subcutaneous region near the tail base) (Fig. 3-a). Results demonstrated that the injection of MLT solution at ST36 induced a rapid increase of fluorescence intensity at 2-h time point, followed by a quick decrease. In comparison, MLT-Gel injection at ST36 induced sustained accumulation of fluorescence, peaking after 24 h, with the signal persisting for more than 96 h (Table S5, Fig. 3-b, d). Furthermore, the acupoint MLT-gel injection consistently generated a higher fluorescence intensity signal compared to the non-acupoint MLT-gel injection (Table S6, Fig. 3-c). The tissue near the joints of the RA mice is typically in an acidic environment, which may have triggered the rapid drug release and high concentration accumulation of CS/GP/HA/MLT-Gel. Conversely, the neutral environment in the subcutaneous of the tail did not activate drug release as effectively, further indicating that the acidic environment at the injection site significantly impacts drug release. The in vivo experimental results are consistent with the in vitro release experiments, suggesting that CS/GP/HA/MLT-Gel possesses pH-responsive properties.

Next, we evaluated fluorescence intensity in major organs and our results demonstrated that the injection of MLT solution at ST36 induced a rapid MLT accumulation in the liver, lungs, and kidneys, reaching the peak levels at the 8-h time point. At the same time, MLT-Gel ST36 administration induced a lower fluorescence intensity in major organs within the first 8 h, reaching the peak at 48 h (Fig. 3-c, Figure S1). These results indicated that MLT-Gel prolonged the systemic circulation time of MLT, reducing hepatic and renal drug metabolism burden and, thus, reducing the toxicity of MLT. To further investigate whether MLT-gel affects liver, lung, and kidney functions, we supplemented the study with serological markers for acute injury in these organs. Changes in alanine aminotransferase (ALT), aspartate aminotransferase (AST), blood urea nitrogen (BUN), creatinine (CR), surfactant protein A (SP-A), and surfactant protein D (SP-D) were analyzed before and after administrations [23]. The results showed that after injecting MLT and MLT-gel, there was a slight increase in serum levels of ALT, CR, SP-A, and SP-D, but no significant differences were observed (Fig. 3-e). This indicates that MLT has a minimal impact on liver, lung, and kidney functions, demonstrating acceptable biosafety with no significant toxicity or damage.

Since one of the primary goals of HC-needle design was to achieve the precise transportation of the composite hydrogel to specific acupoints, we evaluated the ability of HC-needle to transport the hydrogel into the muscle tissue using an ex-vivo porcine hindlimb muscle tissue model. As demonstrated in Fig. 2-f, we hypothesized that the hollow tube and honeycomb pore structure of the HC-needle would effectively protect the hydrogel until it would be expelled upon insertion of the inner needle core, successfully delivering the hydrogel to the desired location. Upon needle insertion, the hydrogel would be released from the honeycomb-like pores in response to the acupoint stimulation, until all of the gel is removed (Fig. 3-g). In contrast, the hydrogel attached to the CA-needle would be blocked at the skin surface during needle insertion into the muscle tissue (Fig. 3-h). During the ex-vivo experiments, both CA- and HC-needles were loaded with Cy-5.5-labelled MLT-Gel and the porcine muscle tissue was pierced (Fig. 3-i). The fluorescence intensity of the HC-needle decreased by 62.01% compared to that of the initial value, confirming its ability to deliver the hydrogel into the muscle tissue. On the other hand, the fluorescence intensity at the tip of the CA-needle decreased by only 49.30% and the gel remained primarily on the surface of the skin, indicating a minimal transfer of hydrogel into the muscle (Fig. 3-j).

To assess the feasibility of drug delivery using the HC-needle in mice, the pore positions were adjusted to accommodate the animal’s body size, ensuring all pores were situated within the last 3 mm from the needle tip (Fig. 3-k). CS/GP/HA/MLT-Gel was labeled with Cy5.5 dye and 5µL of the hydrogel was loaded into the HC-needle, followed by injection into the ST36 acupoint of mice (Fig. 3-l). Histological sections of the muscle tissue at the acupoint site revealed the distribution of the Cy5.5-labeled hydrogel post-injection (Fig. 3-m). The presence of bright red fluorescence indicates successful permeation of the hydrogel from the HC-needle into the internal muscle tissue, thereby confirming effective delivery of CS/GP/HA/MLT-Gel via the HC-needle.

#### MLT-Gel@HC-EA treatment in vivo inhibits cartilage damage and synovitis

**Fig. 4:**
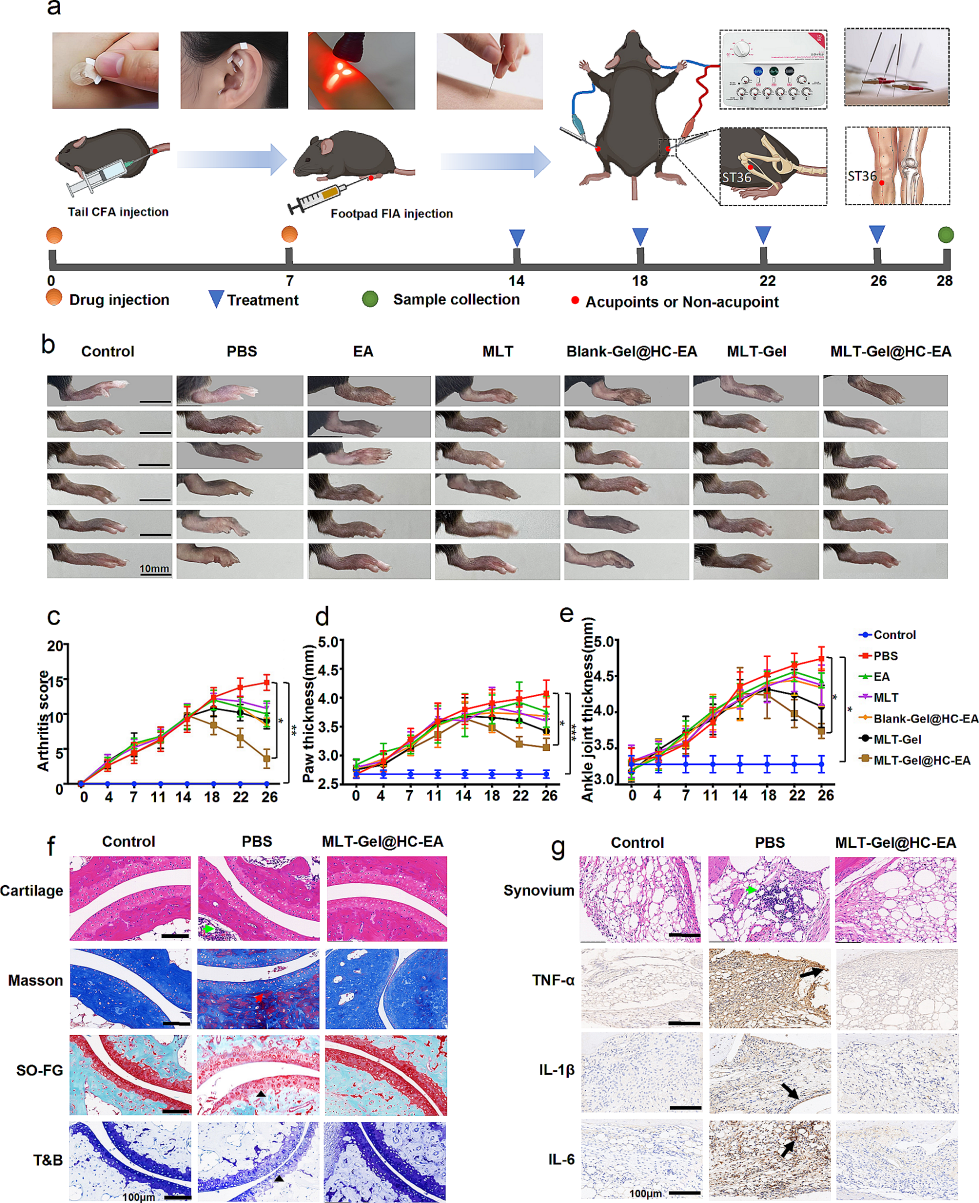
therapeutic efficacy and histopathological analysis. (**a**) schematic of acupuncture and experimental workflow. (**b**) images of lower limbs across different groups. (**c**) arthritic scores of mice in each group. (**d**) comparison of hind paw thickness in each group. (**e**) comparison of ankle joint thickness in each group. (**f**) cartilage staining (he, masson, so-fg, t&b) in various groups. green arrows: invasion of inflammation. red arrows: formation of pannus. black triangle: loss of proteoglycan. scale bars: 100 μm. (**g**) synovial histopathology (he) and immunohistochemistry images in different groups. green arrows: invasion of inflammation. black arrows: positive staining. scale bars: 100 μm

Acupuncture is a therapy that treats diseases by stimulating acupoints with various tools. As time has progressed, acupuncture has evolved into multiple forms (Fig. 4-a). Intradermal acupuncture employs short, thumbtack-shaped needles to stimulate the dermis layer [24]. Ear acupuncture uses medical tape to adhere round plant seeds to acupoints on the ear, which are then stimulated by finger pressure [25]. Laser acupuncture uses fine laser beams to irradiate acupoints [26]. Traditional acupuncture, the most common form, uses stainless steel needles to stimulate the muscle layer at acupoints [27]. To standardize the stimulation parameters of acupuncture, an EA machine with HC-needle was integrated in this study. Based on existing research and in vivo drug experiment results [14, 17], the depth of needle insertion at the ST36 in the hind limbs of mice in this study was determined to be 3–5 millimeters. This depth is conducive to deep muscle stimulation and allows the acid-responsive MLT-Gel to exert its effects in the acidic microenvironment of the joints. On day 14, healthy mice were assigned to a control group (*n* = 6, ST36 PBS injection), while 36 RA model mice were randomly divided into 6 groups (*n* = 6/group): the PBS group (ST36 PBS injection), the EA group (ST36 Electroacupuncture), the MLT group (non-acupoint MLT solution injection), the Blank-Gel@HC-EA group (ST36 Electroacupuncture with Blank-gel), the MLT-Gel group (non-acupoint MLT-Gel injection), and the MLT-Gel@HC-EA group (ST36 Electroacupuncture with MLT-Gel). The treatment was administered every 4 days, with the MLT dosage of 0.15 µg per mouse (Fig. 4-a). As the disease progressed, on day 28, RA mice administered with PBS displayed severe arthritis symptoms compared to the Control group, while mice treated with MLT-Gel@HC-EA showed no visible inflammation (Table S6, Fig. 4-b). Every 4 days, an assessment is conducted on mice for arthritis score, hind paw thickness, and ankle joint thickness. On day 18, the arthritis scores for the treatment groups reached their highest point, followed by a subsequent decline; however, the scores of the PBS group continued to increase (Fig. 4-c, Table S1). Furthermore, the hind paw and ankle joint thickness in mice treated with MLT-Gel@HC-EA appeared to be lower compared to that in all other treatment groups, corresponding to the trends observed in the arthritis score results (Fig. 4-d, e). These results suggested that MLT-Gel@HC-EA reduced local swelling and inflammation compared to using acupuncture or MLT alone, indicating that acupoint and MLT-Gel administration were more effective than non-acupoint and MLT solution administration.

Next, we evaluated the mouse joints using histological staining. Masson’s staining demonstrated that PBS-treated mice had significant articular cartilage erosion and vascular proliferation in the ankle joints. In contrast, the articular surfaces, clear interfaces, and decreased cell infiltration in the MLT-Gel@HC-EA group sections was similar to those of the Control group. Furthermore, Safranin O-fast green (SO-FG) staining showed significant proteoglycan depletion in the joints of the PBS group, indicating severe cartilage impairment; however, the proteoglycan staining in the MLT-Gel@HC-EA group was similar to the Control group mice. In addition, the Toluidine Blue (T&B) staining demonstrated lower glycosaminoglycan levels in the PBS group cartilage compared to the Control group; however, the staining levels in the MLT-Gel@HC-EA group were similar to those in the Control group, suggesting the protective properties of the treatment (Table S7, Fig. 4-f). These histopathological findings indicated that MLT-Gel@HC-EA treatment reduced inflammation and slowed disease progression (Table S2, Figure S2).

To evaluate the expression levels of pro-inflammatory cytokines, such as TNF-α, IL-1β, and IL-6, in the synovial membrane, immunohistochemical staining was performed. The staining intensity levels of TNF-α, IL-1β, and IL-6 appeared to be higher in the PBS group, confirming the presence of these cytokines at the onset of RA (Fig. 4-g). At the same time, the staining intensity of these cytokines appeared to be lower in the MLT-Gel@HC-EA group (Table S7, Figure S3). These results further confirmed our histopathological findings, including reduced synovial inflammation and cartilage erosion. To evaluate the MLT-Gel safety profile, we performed histopathology on major mouse organs, such as the heart, liver, spleen, lungs, and kidneys. Minimal toxicity was observed in all major organs for both acupuncture and MLT-Gel treatments; however, the MLT solution injection induced mouse myocardial cell toxicity, leading to cell damage. These toxicity and adverse effects induced by the MLT solution were not observed in the MLT-Gel@HC-EA group, confirming the overall safety of the treatment (Figure S4).

#### Network pharmacology analysis of potential targets

**Fig. 5:**
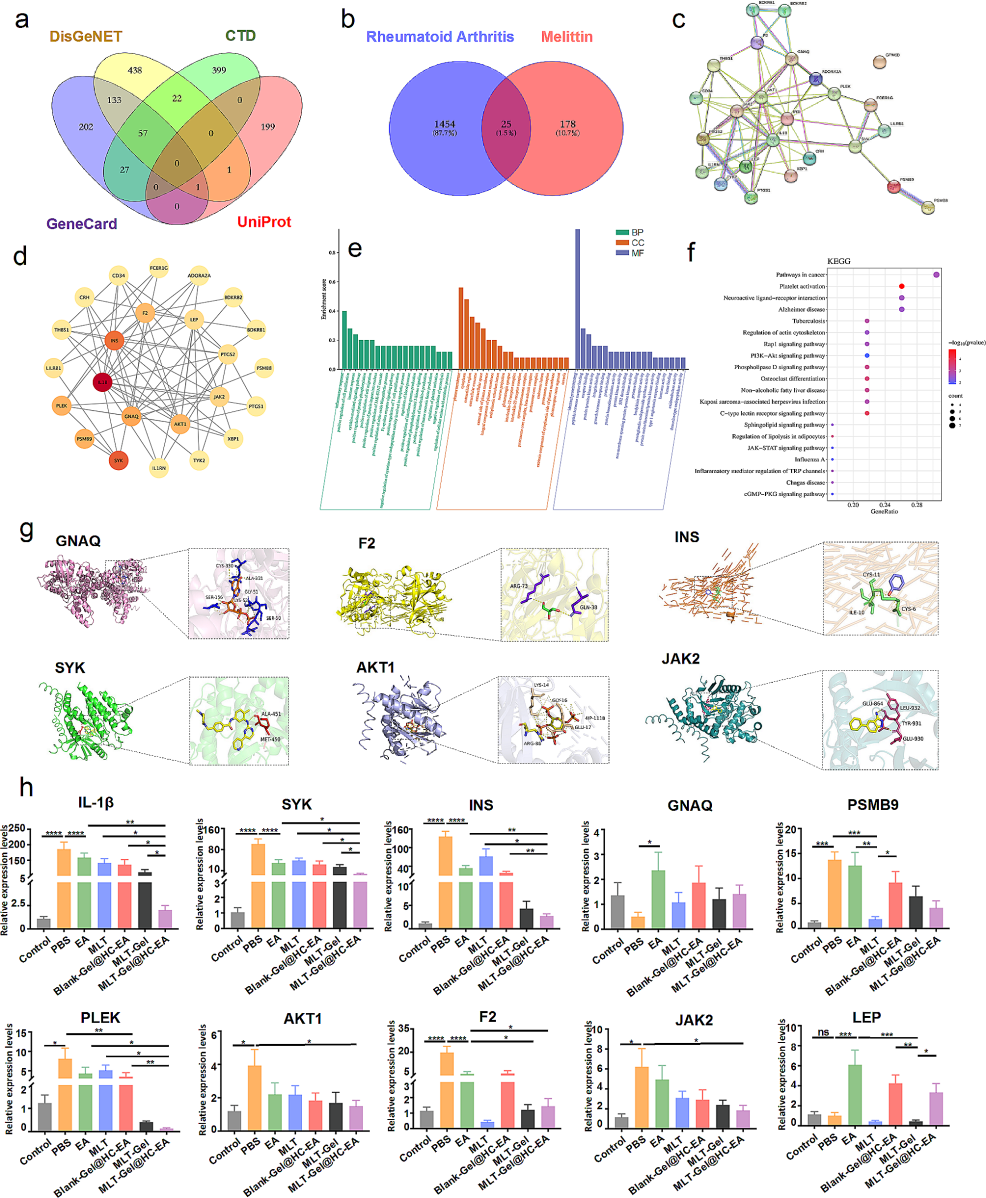
network pharmacology analysis of core targets between mlt and ra. (**a**) venn diagram of associated targets in various gene libraries. (**b**) venn diagram of overlapping genes between mlt and ra. (**c**) protein-protein interaction (ppi) network. (**d**) core gene network map. (**e**) bar graphs for go analysis (bp, cc, mf). (**f**) enrichment pathway analysis by kegg. (**g**) molecular docking map of core targets between MLT and RA. (**h**) The relative expression levels of the top 10 targets. (^*^P < 0.05, ^**^P < 0.01, ^***^P < 0.001, ^****^P < 0.0001)

To elucidate the potential mechanism of MLT-Gel action, we performed network pharmacology analysis. The analysis of several databases determined 1454 RA targets and 178 MLT targets (Fig. 5-a), while intersection analysis identified 25 shared targets (Fig. 5-b). Subsequently, 10 core targets, including IL-1β, AKT1, JAK2, and SKY, were selected, indicating their pivotal role in the RA treatment by MLT (Fig. 5-c, d). The Gene Ontology (GO) term analysis demonstrated the involvement of biological processes (BP), such as inflammatory response, positive regulation of cell proliferation, and immune response; cellular components (CC), such as plasma membrane, cytoplasm, and extracellular region; and molecular functions (MF), such as identical protein binding, peptide hormone receptor, and protein tyrosine kinase activity (Fig. 5-e). Kyoto Encyclopedia of Genes and Genomes (KEGG) analysis was used to identify the primary pathways activated during the MLT treatment of RA. These pathways were associated with cancer, neuroactive ligand-receptor interaction, and osteoclast differentiation, indicating potential approaches for therapeutic intervention (Fig. 5-f). In addition, molecular docking energy analysis provided information regarding the binding activity between MLT and core target proteins. Among the selected targets, GNAQ (-16.3), F2 (-14.0), INS (-13.9), SYK (-12.3), AKT (-11.0), and JAK2 (-6.9) exhibited binding energies all < -5 kcal/mol, suggesting robust binding activity (Fig. 5-g). The bioinformatics analysis results provided us with some high-frequency targets. To further validate the sensitivity of EA and MLT to these targets, we collected ankle joint tissues from 28-day RA mice and used qPCR to detect the expression levels of the top 10 targets (Fig. 5-h). The analysis results indicated that both EA and MLT reduced the levels of IL-1β, SYK, INS, PLEK, AKT1, F2, and JAK2 in RA model mice. However, for PSMB9 and LEP, the regulatory directions of EA and MLT were opposite. The synergistic downregulation of IL-1β, SYK, INS, PLEK, AKT1, F2, and JAK2 by EA and MLT suggests that these might be the action targets of MLT-Gel@HC-EA. IL-1β, SYK, INS, PLEK, AKT1, F2, and JAK2 are mainly associated with the NF-κB and AKT pathways. These findings suggest that the MLT-Gel@HC-EA treatment for RA involves the regulation of multiple targets, providing insights for our subsequent focus on inflammation pathways.

#### MLT-Gel@HC-EA treatment regulates immune cell function

**Fig. 6:**
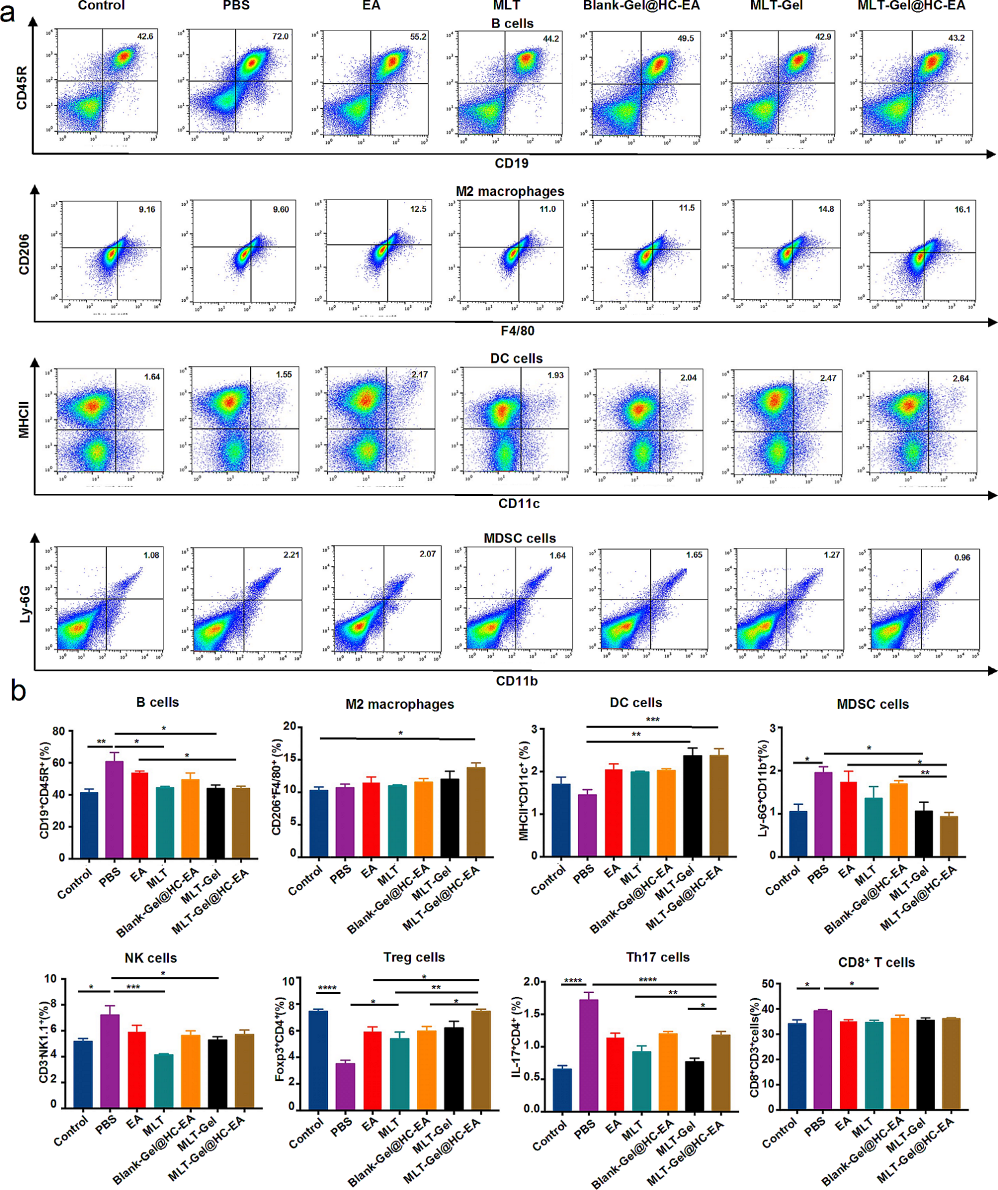
Flow cytometry analysis of immune cells. (**a**) Representative scatter plots of immune cells. (**b**) Comparison of immune cell ratio among different groups. (^*^P < 0.05, ^**^P < 0.01, ^***^P < 0.001, ^****^P < 0.0001)

Based on the network pharmacology results, we evaluated the effect of MLT-Gel@HC-EA treatment on immune cell function. Flow cytometry analysis of mouse spleen cells revealed elevated levels of B cells, myeloid-derived suppressor cells (MDSCs), NK cells, Th17 cells, and CD8^+^ T cells in the PBS group. In contrast, the MLT-Gel@HC-EA group exhibited increased levels of M2 macrophages, Treg cells, and DC cells compared to the PBS group (Table S8, Fig. 6). Increased number of B cells can enhance the activation and proliferation of T cells, resulting in local inflammatory cell infiltration, while activated NK cells could interact with MDSCs, leading to bone and cartilage destruction. Although MDSCs can demonstrate some immunosuppressive effects, they also secrete numerous inflammatory factors and activate TH17 cells, leading to the production of IL-17 A (Fig. 6, Figure S5). At the same time, MLT-Gel@HC-EA appeared to increase the number of M2 macrophages, suppressing the release of inflammatory cytokines. Furthermore, MLT-Gel@HC-EA appeared to stimulate DC cells to induce tolerance in T cells, regulating the expansion of Treg cells and restoring the Th17/Treg equilibrium. Not all immune cell populations exhibit significant differences, but these trends are still biologically meaningful. In this study, besides M2 macrophages and CD8 + T cells, other immune cells also showed some modulation. The increase in Treg cells and DC cells, along with the decrease in B cells, MDSCs, and TH17 cells, may collectively contribute to therapeutic effects. Despite the lack of significant changes in some immune cells, these comprehensive regulatory effects support MLT-Gel@HC-EA, and their modulation should be considered as part of a broader immunomodulatory context.

### MLT-Gel@HC-EA inhibits inflammatory factors and downregulates inflammatory pathways

**Fig. 7:**
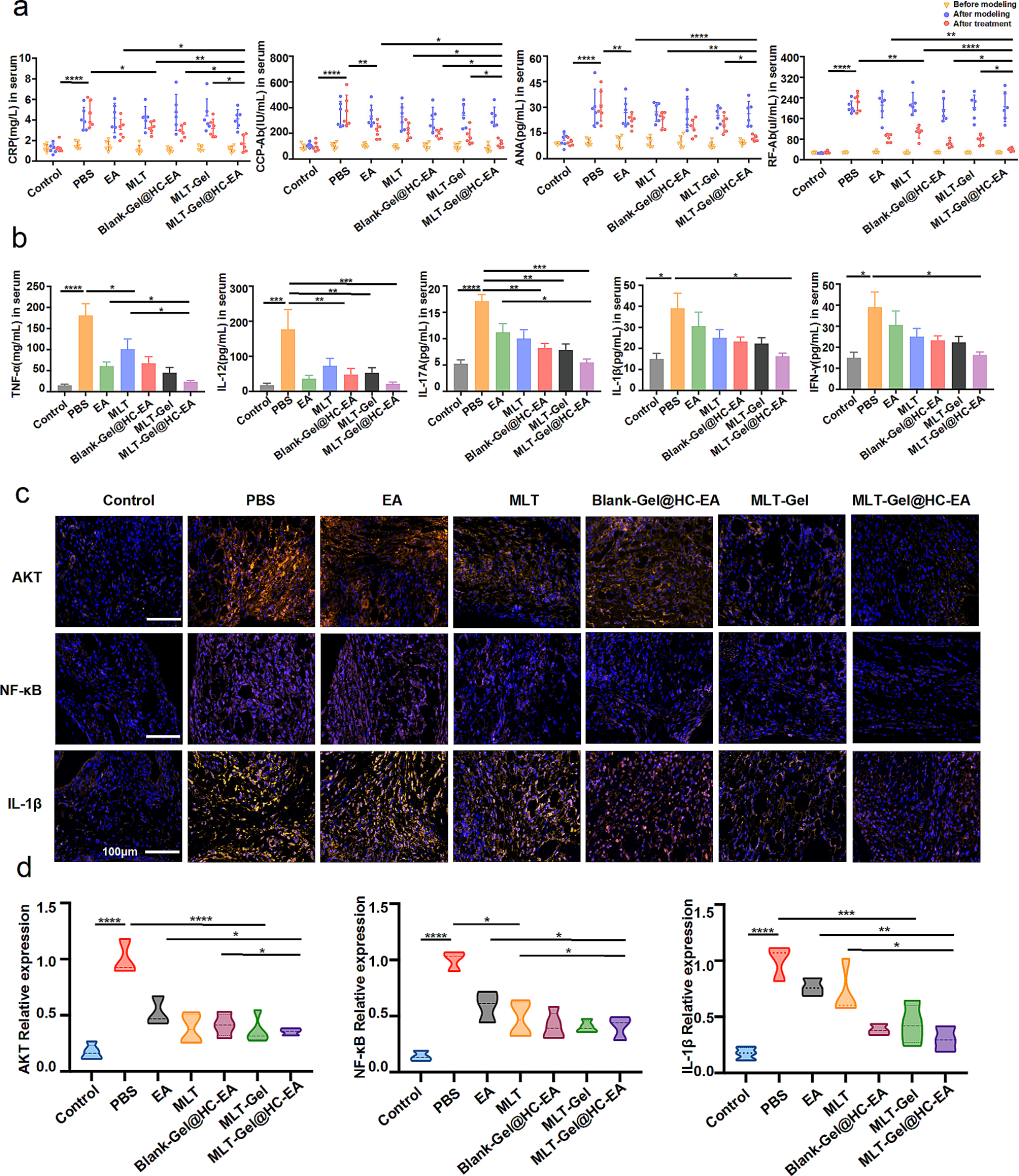
Analysis of of inflammatory pathways. (**a**) the levels of ra biomarkers in serum (crp, ccp-ab, ana, and rf-ab). (*p < 0.05, **p < 0.01, ***p < 0.001, ****p < 0.0001). (**b**) Serum concentration of pro-inflammatory cytokines (tnf-α、il-12、il-1β、ifn-γ andil-17a). (*p < 0.05, **p < 0.01, ***p < 0.001, ****p < 0.0001). (**c**) if confirmed the expression level of akt, nf-κb and il-1β. Scale bars: 100 μm. (**d**) comparison of if expression levels among different groups. (^*^p < 0.05, ^**^p < 0.01, ^***^p < 0.001, ^****^p < 0.0001)

In previous studies, the success of RA modeling was determined by visually observing the local manifestations of redness, swelling, and heat in the animal’s joints, and reaching a certain arthritis score [28]. To provide more convincing data, we referred to some serological markers required for the clinical diagnosis of RA [29, 30]. The results showed that after modeling, the levels of RA biomarkers C-reactive protein (CRP), cyclic citrullinated peptide antibody (CCP-Ab), antinuclear antibody (ANA), and rheumatoid factor antibody (RF-Ab) were significantly elevated compared to pre-modeling levels, and decreased after treatment (Fig. 7-a). This indicates that the CFA/IFA method is suitable for the RA model and exhibits similarities to clinical RA patients. MLT-Gel@HC-EA significantly reduced the levels of RA biomarkers and alleviated the progression of RA in mice compared to other treatment groups. Using bioinformatics analysis, several inflammatory factors and their associated signaling pathways were identified. To investigate the effect of MLT-Gel treatment on the levels of pro-inflammatory cytokines in the serum of RA mice, TNF-α, IL-12, IL-1β, Interferon-γ (IFN-γ), and Interleukin-17 A (IL-17 A) concentrations were measured using ELISA kits. Our findings demonstrated that both acupuncture and MLT significantly inhibited the expression of pro-inflammatory cytokines. These findings suggested a potential for synergy in the anti-inflammatory effects of this integrated drug delivery system (Fig. 7-b). To elucidate the underlying mechanisms of MLT-Gel@HC-EA treatment in RA, we investigated the NF-κB, AKT, and IL-1β expression levels using immunofluorescence (Table S9, Fig. 7-c, Figure S5). The results indicated a significant reduction of fluorescence intensity levels of all three proteins in response to MLT-Gel@HC-EA treatment compared to the PBS group, demonstrating its ability to alleviate RA inflammation by modulating immune pathways (Fig. 7- d).

## Discussion

Synovial inflammation is one of the fundamental and crucial pathological characteristics of RA. In patients with RA, the synovial fluid of the joint contains considerable inflammatory cell infiltration. Within the inflamed synovial tissue, a typical synovial lining of 2–3 layers transform into a highly proliferative synovial cell structure consisting of 10–20 layers. This leads to the formation of vascular pannus structures that attach to joint cartilage surfaces [31], resulting in erosion and degradation of the cartilage and joint structure deterioration. Synovial hyperplasia, immune cell infiltration, and elevated expression of various pro-inflammatory cytokines collectively increase the oxygen demand of the synovium, resulting in a hypoxic microenvironment in RA [32, 33]. RA induced by complete Freund’s adjuvant (CFA) and collagen injection is a commonly-used animal model that induces autoimmune responses in mice, generating antibodies against type II collagen and resulting in the onset of inflammatory arthritis, simulating pathological features observed in patients with RA. Furthermore, it has been reported that the pH levels of healthy synovial fluid and arthritic synovial fluid range between 7.4 and 7.8 and 6.6–7.2, respectively [14]. The presence of acidic microenvironment in the affected joints needs to be accounted for when designing novel therapeutic strategies and modifying inflammatory conditions.

CS is a polycationic dimer with excellent histocompatibility, biodegradability, and adhesive properties. Its usage in medical biology has been the subject of extensive research [34]. The formation of CS/GP-Gel is primarily attributed to the hydrogen bonding and hydrophobic interactions between the molecular chains of CS and β-GP. CS/GP-Gel is commonly used for drug delivery, since this gel remains in a liquid form at room temperature and transform into a gel form at temperatures up to 37 °C [20]. HA is a naturally occurring acidic mucopolysaccharide commonly present in cartilage matrix and joint fluids. Due to its excellent biocompatibility, HA is extensively used for the treatment of cartilage-related pathologies [35]. HA contains a significant number of amino and carboxyl groups, and its structure depends on the pH of the solution [36]. In a neutral setting, in the presence of EDC/NHS, HA binds with CS/GP-Gel to generate a composite hydrogel. In an acidic environment, the ionization of carboxyl groups of HA increases, elevating its negative charge density [21]. As a result, the hydrogel network disintegrates, enabling the release of MLT from the hydrogel. This ability to degrade in an acidic environment, which corresponds to the weakly acidic microenvironment of RA, allows the passive targeting of CS/GP/HA/MLT-Gel.

Compared to the (healthy) Control group, the mice in the RA PBS group demonstrated elevated levels of B cells, MDSCs, NK cells, Th17 cells, and CD8^+^ cells, as well as reduced levels of M2 macrophages, Treg cells, and DC cells. Earlier research has shown that during the initial stages of RA, B cells become activated and differentiate into plasma cells, producing high amounts of immunoglobulins, which combine with rheumatoid factors to cause inflammation [37] In addition, B cells activate helper T cells by presenting self-antigens to CD4^+^ T cells. As the disease progresses, B cells infiltrate the synovial tissue, resulting in the formation of an intra-synovial inflammatory microenvironment that initiates a prolonged and detrimental autoimmune response [38]. MDSCs have T-cell immunosuppressive properties; however, they can also release high levels of inflammatory cytokines, including IL-1β and TNF-α [39]. In the peripheral blood of RA patients, MDSCs undergo significant amplification, which positively correlates with disease severity and the responsiveness of inflammatory Th17 cells. Moreover, in vitro experiments indicate MDSCs induce Th17 cell polarization in mice [40]. Another study reported the presence of NK cells in inflamed synovial sites of patients with RA at the early stages of the disease, co-existing with MDSCs [41]. The established hypothesis suggests that NK cells may interact with MDSCs and induce their differentiation into bone-resorbing osteoclasts, ultimately resulting in the destruction of bone and cartilage.

DCs, which are considered one of the most competent antigen-presenting cells (APCs), regulate adaptive immune responses depending on their specific microenvironment. DCs possess both stimulatory and suppressive properties, and can regulate immune responses by presenting surface antigen peptides, MHC II, CD80, and CD86 co-stimulatory molecules, ultimately activating T cells and inducing their interactions [42]. As immunosuppressive cells, DCs regulate the subsets of activated CD4^+^ T cells, also known as Tregs. In contrast to T follicular helper cells (Tfh), which facilitate the inflammatory response, Tregs are highly efficient at suppressing RA. Once activated, their immunosuppressive effects are not specific to antigens and can inhibit both the activation and proliferation of CD4^+^ T and CD8^+^ T cells [43]. In addition, Tregs downregulate the proliferation of NK cells and induce cytotoxic effects, leading to cell death in several autologous target cells, including macrophages and B cells [44]. The regulation of macrophage polarization plays a crucial role in maintaining immune homeostasis, promoting tissue repair, and facilitating the progression of diseases. Inducing activation of anti-inflammatory M2 macrophages allows to decrease the production of inflammatory cytokines, thereby alleviating RA synovitis. IL-1β, TNF-α, and IL-6 are the primary pro-inflammatory cytokines linked to RA. They can trigger activation of several inflammatory pathways, including NF-κB and AKT, which induce the differentiation of osteoclasts [45]. The KEGG enrichment analysis indicated that the main targets of MLT and RA were involved in several pathways, such as cancer, neuroactive ligand-receptor interaction, osteoclast differentiation, PI3K-AKT signaling, JAK-STAT signaling, C-type lectin receptor signaling, and TRP pathways. All of these pathways are intricately related to autoimmunity and inflammation. In summary, the interaction between immune cells and inflammatory factors in the arthritic microenvironment is critical for the pathogenesis and progression of RA. Our study indicated that MLT-Gel@HC-EA activated M2 macrophages, suppressed inflammatory cytokine secretion, and induced immune tolerance in T cells via DC cells. In addition, MLT-Gel@HC-EA increased Treg cell proliferation, restoring the Th17/Treg ratio. The inhibitory effect of MLT-Gel@HC-EA on inflammatory reactions could be due to the downregulation of AKT and NF-κB signaling pathways. Future studies should examine the mechanism of MLT-Gel@HC-EA action upstream of AKT and NF-κB, thus identifying signaling pathways involved in the regulation of inflammation.

The drug delivery system MLT-Gel@HC-EA consists of two components. The use of MLT-Gel alone or EA alone can both alleviate RA. MLT-Gel can be delivered with the help of other tools, and the acupuncture delivery platform can also deliver other drugs. Both have broad application prospects. Due to the convergence of their regulatory mechanisms, they can achieve the best comprehensive effect when used in combination. It is noteworthy that traditional acupuncture usually involves multiple stainless steel needles treating different acupoints. The stimulation of different acupoints may produce complex network effects, making it difficult to determine the specific mechanisms of action. To facilitate the observation of the drug delivery system MLT-Gel@HC-EA in this study and to eliminate the influence of multiple acupoints, we temporarily performed acupuncture stimulation only at a single acupoint, ST36. When MLT-Gel is administered subcutaneously, both the metabolism duration and the drug accumulation concentration are affected. The overall efficacy of the MLT-Gel group, which received subcutaneous injections in the tail, was inferior to that of the MLT-Gel@HC-EA group. Therefore, it can be inferred that the depth of injection influences the final efficacy of electroacupuncture. Consequently, we adopted the method of inserting the HC-needle into muscle tissue in this study. The HC-needle played a dual role in this study, serving both as a tool for drug delivery and as a therapy that can directly modulate RA immune function. This hollow, drug-containing modified acupuncture needle has the potential to improve the experience of traditional acupuncture in the future and to play a broader role in combination with drugs.

In conclusion, our study provides evidence for the potential role of MLT-Gel@HC-EA in the treatment of RA synovitis and reduction of joint destruction. Our findings suggest that this formulation shows promising results as a new targeted therapeutic agent for the RA treatment.

### Electronic supplementary material

Below is the link to the electronic supplementary material.

Supplementary Material 1

Supplementary Material 2

## Data Availability

The data that support the findings of this study are available on request from the corresponding author.

## Abbreviations

RA: Rheumatoid Arthritis
CA: Classical Acupuncture
EA: Electroacupuncture
MLT: Melittin
OA: Osteoarthritis
CS: Chitosan
β-GP: β-Glycerophosphate
HA: Hyaluronic Acid
HC: Honeycomb
NK: Natural Killer
DC: Dendritic Cell
Treg: Regulatory T
TNF-α: Tumor Necrosis Factor-α
IL-6: Interleukin-6
IL-β: Interleukin-β
EDC: N-(3-Dimethylaminopropyl)-N′-Ethylcarbodiimide Hydrochloride
NHS: N-Hydroxysuccinimide
ST36: Zusanli
ALT: Alanine Aminotransferase
AST: Aspartate Aminotransferase
BUN: Blood Urea Nitrogen
CR: Creatinine
SP-A: Surfactant Protein A
SP-D: Surfactant Protein D
GO: Gene Ontology
BP: Biological Processes
CC: Cellular Components
MF: Molecular Functions
KEGG: Genes and Genomes
MDSCs: myeloid-derived Suppressor Cells
CRP: C-Reactive Protein
CCP-Ab: Cyclic Citrullinated Peptide Antibody
ANA: Antinuclear Antibody
RF-Ab: Rheumatoid Factor Antibody
IFN-γ: Interferon-γ
IL-17A: Interleukin-17 A
IF: Immunofluorescence
