# Supplementary material for: The combination of modified acupuncture needle and melittin hydrogel as a novel therapeutic approach for rheumatoid arthritis treatment
Source: J Nanobiotechnology. 2024 Jul 22;22:432. doi: 10.1186/s12951-024-02722-y (PMC11265141; doi:10.1186/s12951-024-02722-y)
Supplement: Supplementary file 1 — Supplementary Material 1 [file 12951_2024_2722_MOESM1_ESM.docx]

**Supporting file 1**


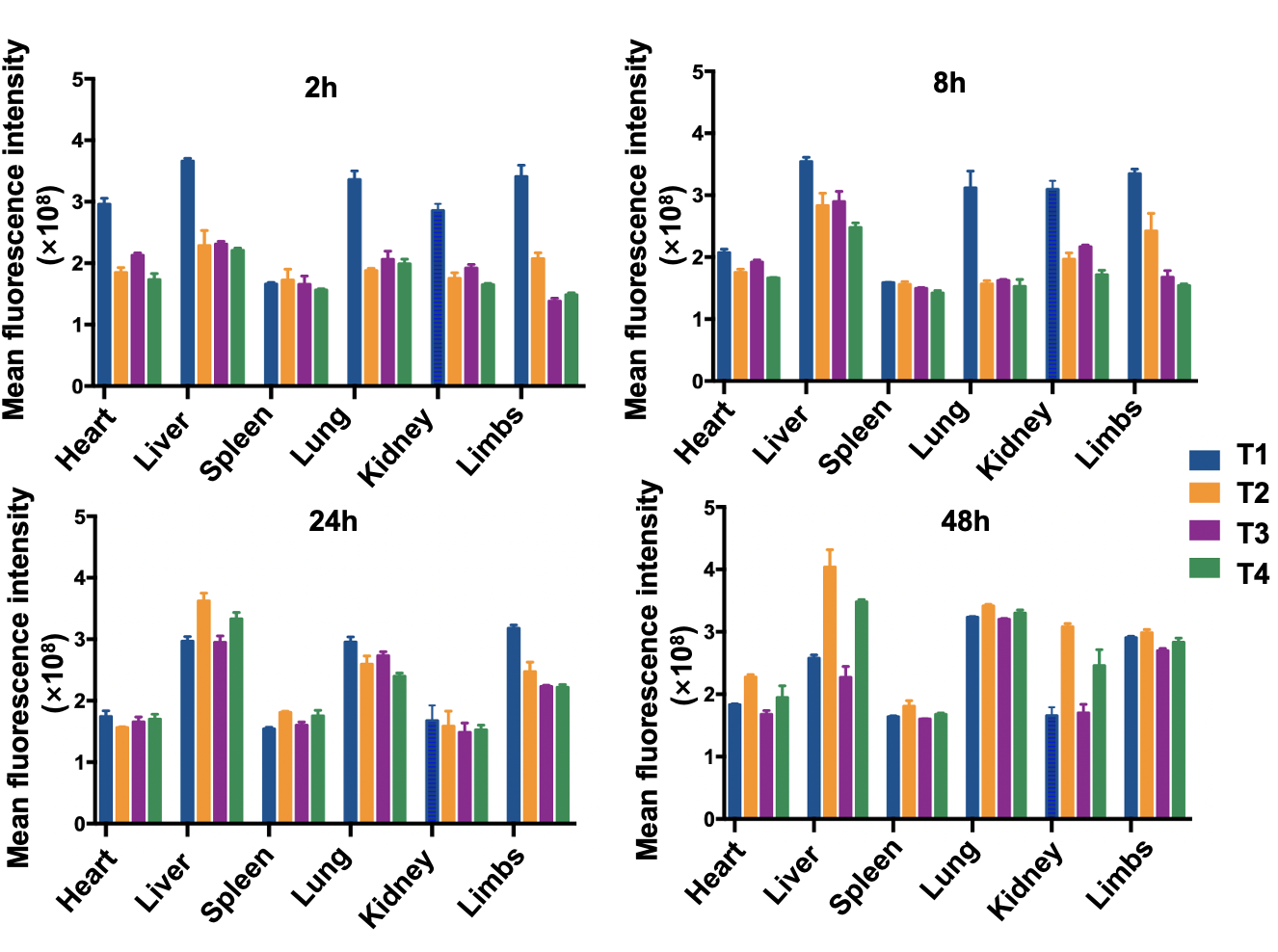


Figure S1. Quantitative analysis of fluorescence in different organs at different time.

 Figure S2. Cartilage staining (HE, Masson, SO-FG, T&B) in various groups. a) Histological sections with H&E, Masson, SO-FG and T&B staining of joints in different groups. Green arrows: invasion of inflammation. Red arrows: formation of pannus. Black triangle: loss of proteoglycan. Scale bar: 100 μm. b) Histopathologic scores of synovial inflammation, bone erosion, and cartilage degradation. (^*^*P* < 0.05, ^**^*P* < 0.01, ^***^*P* < 0.001, ^****^*P* < 0.0001).

Figure S3: Immunohistochemistry analysis of articular tissues. a) immunohistochemical image of cartilage and synovium in different groups. Black arrows: positive staining. Scale bar:100 μm. b) Quantification of the TNF-α, IL-1β and IL-6 production in the tissues treated with different samples based on the immunohistochemical results. (^*^*P* < 0.05, ^**^*P* < 0.01).

Figure S4. H&E-stained images of major organs (heart, liver, spleen, lungs, and kidneys), the red arrow represents myocardial cell rupture. Scale bars: 100 μm.

Figure S5. Representative scatter plots of immune cells.

Figure S6. Immunofluorescence images of synovium. a) Fluorescent expression of AKT in synovium. b) Fluorescent expression of NF-κB in synovium. c) Fluorescent expression of IL-1β in synovium.
