# Supplementary material for: The combination of modified acupuncture needle and melittin hydrogel as a novel therapeutic approach for rheumatoid arthritis treatment
Source: J Nanobiotechnology. 2024 Jul 22;22:432. doi: 10.1186/s12951-024-02722-y (PMC11265141; doi:10.1186/s12951-024-02722-y)
Supplement: Supplementary file 2 — Supplementary Material 2 [file 12951_2024_2722_MOESM2_ESM.docx]

**Supporting file 2**

Table S1. Arthritis Scores

| Grade | Symptoms | Score |
| --- | --- | --- |
| 0 | No apparent joint swelling or redness | 0 |
| 1 | Mild joint swelling and/or redness | 1 |
| 2 | Significant joint swelling and/or redness | 2 |
| 3 | Severe joint swelling, redness, and/or deformity | 3 |
| 4 | Severe joint swelling, redness, deformity, and impaired function | 4 |

Table S2. Histopathological scores

|  | Features | Score |
| --- | --- | --- |
| Bone erosion | Absent | 0 |
|  | Minimal (small areas of resorption, not readily apparent on low magnification, in the distal tibial trabecular or cortical bone, rare osteoclasts) | 1 |
|  | Mid (more numerous areas of resorption, not readily apparent on low magnification, in the distal tibial trabecular or cortical bone, more numerous osteoclasts) | 2 |
|  | Moderate (obvious resorption of the medullary trabecular and cortical bone, without full-thickness defects in the cortex; loss of some medullary trabeculae, more numerous osteoclasts) | 3 |
|  | Marked (full-thickness defect in the cortical bone, often with distortion of the profile of the remaining cortical surface; marked loss of the medullary bone of the distal tibia; numerous osteoclasts; no resorption in the smaller tarsal bones) | 4 |
|  | Severe (full-thickness defects in the cortical bone, often with distortion of the profile of the remaining cortical surface; marked loss of the medullary bone of the distal tibia, numerous osteoclasts; resorption also present in the smaller tarsal bones) | 5 |
| Inflammation | Absent | 0 |
|  | Minimal infiltration of inflammatory cells in periarticular tissue | 1 |
|  | Mild infiltration | 2 |
|  | Moderate infiltration, with moderate edema | 3 |
|  | Marked infiltration, with marked edema | 4 |
|  | Severe infiltration, with severe edema | 5 |
| Cartilage damage | Absent | 0 |
|  | Minimal (minimal-to-mild loss of cartilage evident on toluidine blue staining, with no obvious chondrocyte loss or collagen disruption) | 1 |
|  | Mild (mild loss of cartilage on toluidine blue staining, with mild focal (superficial) chondrocyte loss and/or collagen disruption) | 2 |
|  | Moderate (moderate loss of cartilage on toluidine blue staining, with moderate multifocal (depth to middle zone) chondrocyte loss and/or collagen disruption) | 3 |
|  | Marked (marked loss of cartilage on toluidine blue staining, with marked multifocal (depth to deep zone) chondrocyte loss and/or collagen disruption) | 4 |
|  | Severe (severe diffuse loss of cartilage on toluidine blue staining, with severe multifocal (depth to tidemark) chondrocyte loss and/or collagen disruption) | 5 |

Table S3. All the primers used in the qPCR analysis

| ID | Gene | Primer | Sequence |  |
| --- | --- | --- | --- | --- |
| 1 | β-actin | forward sequence | 5′-CCATCGAGCACGGCATC-3’ |  |
|  |  | reverse sequence | 5′-ATTGTAGAAGGTGTGGTGCAGA-3’ |  |
| 2 | IL-1β | forward sequence | 5′-ATGATGGCTTATTACAGTGGCAA-3′ |  |
|  |  | reverse sequence | 5′-GTCGGAGATTCGTAGCTGGA-3′ |  |
| 3 | SYK | forward sequence | 5′-GAAGCCTTGCTAAGTGCGACA-3′ |  |
|  |  | reverse sequence | 5′-AAGTGCCGTGAATGGGTGAC-3′ |  |
| 4 | INS | forward sequence | 5′-CTGGTGGGCATCCAGTAACC-3′ |  |
|  |  | reverse sequence | 5′-CATGTTGAAACAATGACCTGCT-3′ |  |
| 5 | GNAQ | forward sequence | 5′-CCCAGAATATGATGGACCCCA-3′ |  |
|  |  | reverse | 5′-ATGTTCTCGGTATCTGTGGCG-3′ |  |
|  |  |  |  |  |
| 6 | PSMB9 | forward sequence | 5′-TCCACACCGGGACAACCAT-3′ |  |
|  |  | reverse sequence | 5′-AACCTGAGAGGGCACAGAAGA-3′ |  |
| 7 | PLEK | forward sequence | 5′-CTCACGGACTGGGAAATGAGG-3′ |  |
|  |  | reverse sequence | 5′-CCCCAAAAGCATGTGAGGAAG-3′ |  |
| 8 | AKT1 | forward sequence | 5′-GGACTACTTGCACTCCGAGAAG-3′ |  |
|  |  | reverse sequence | 5′-CATAGTGGCACCGTCCTTGATC-3′ |  |
| 9 | F2 | forward sequence | 5′-GACCTTTTGTCATGAAGAGCCC-3′ |  |
|  |  | reverse sequence | 5′-GCGTGTAGAAGCCGTATTTCC-3′ |  |
| 10 | JAK2 | forward sequence | 5′-GCTACCAGATGGAAACTGTGCG-3′ |  |
|  |  | reverse sequence | 5′-GCCTCTGTAATGTTGGTGAGATC-3′ |  |
| 11 | LEP | forward sequence | 5′-AGCTGCAAGGTGCAAGAAGAA-3′ |  |
|  |  | reverse sequence | 5′-GGAATGAAGTCCAAGCCAGTGAC-3′ |  |

Table S4. The sample numbers in Figure 3b

| Time  Group | 0h | 2h | 8h | 12h | 24h | 48h | 72h | 96h |
| --- | --- | --- | --- | --- | --- | --- | --- | --- |
| T1 | T1-4 | T1-4 | T1-4 | T1-4 | T1-4 | T1-4 | T1-4 | T1-4 |
| T2 | T2-4 | T2-4 | T2-4 | T2-4 | T2-4 | T2-4 | T2-4 | T2-4 |
| T3 | T3-4 | T3-4 | T3-4 | T3-4 | T3-4 | T3-4 | T3-4 | T3-4 |
| T4 | T4-4 | T4-4 | T4-4 | T4-4 | T4-4 | T4-4 | T4-4 | T4-4 |

Table S5. The sample numbers in Figure 3b

| Time  Group | 2h | 8h | 24h | 48h |
| --- | --- | --- | --- | --- |
| T1 | T1-1 | T1-2 | T1-3 | T1-4 |
| T2 | T2-1 | T2-2 | T2-3 | T2-4 |
| T3 | T3-1 | T3-2 | T3-3 | T3-4 |
| T4 | T4-1 | T4-2 | T4-3 | T4-4 |

Table S6. The sample numbers in Figure 4b

| Control | PBS | EA | MLT | Blank-Gel@HC-EA | MLT-Gel | MLT-Gel@HC-EA |
| --- | --- | --- | --- | --- | --- | --- |
| Control-1 | PBS-1 | EA-1 | MLT-1 | Blank-Gel@HC-EA-1 | MLT-Gel-1 | MLT-Gel@HC-EA-1 |
| Control-2 | PBS-2 | EA-2 | MLT-2 | Blank-Gel@HC-EA-2 | MLT-Gel-2 | MLT-Gel@HC-EA-2 |
| Control-3 | PBS-3 | EA-3 | MLT-3 | Blank-Gel@HC-EA-3 | MLT-Gel-3 | MLT-Gel@HC-EA-3 |
| Control-4 | PBS-4 | EA-4 | MLT-4 | Blank-Gel@HC-EA-4 | MLT-Gel-4 | MLT-Gel@HC-EA-4 |
| Control-5 | PBS-5 | EA-5 | MLT-5 | Blank-Gel@HC-EA-5 | MLT-Gel-5 | MLT-Gel@HC-EA-5 |
| Control-6 | PBS-6 | EA-6 | MLT-6 | Blank-Gel@HC-EA-6 | MLT-Gel-6 | MLT-Gel@HC-EA-6 |

Table S7. The sample numbers in Figure 4f-g

| Group  Index | Control | PBS | MLT-Gel@HC-EA | Group  Index | Control | PBS | MLT-Gel@HC-EA |
| --- | --- | --- | --- | --- | --- | --- | --- |
| T1 | Control-1 | PBS-1 | MLT-Gel@HC-EA-1 | T1-4 | Control-1 | PBS-1 | MLT-Gel@HC-EA-1 |
| T2 | Control-1 | PBS-1 | MLT-Gel@HC-EA-1 | T2-4 | Control-1 | PBS-1 | MLT-Gel@HC-EA-1 |
| T3 | Control-1 | PBS-1 | MLT-Gel@HC-EA-1 | T3-4 | Control-1 | PBS-1 | MLT-Gel@HC-EA-1 |

Table S8. The sample numbers in Figure 6b

| Group  Index | Control | PBS | EA | MLT | Blank-Gel@HC-EA | MLT-Gel | MLT-Gel@HC-EA |
| --- | --- | --- | --- | --- | --- | --- | --- |
| B cells | Control-1 | PBS-4 | EA-1 | MLT-2 | Blank-Gel@HC-EA-1 | MLT-Gel-1 | MLT-Gel@HC-EA-4 |
| M2 macrophages | Control-2 | PBS-2 | EA-5 | MLT-4 | Blank-Gel@HC-EA-3 | MLT-Gel-2 | MLT-Gel@HC-EA-4 |
| DC cells | Control-5 | PBS-5 | EA-4 | MLT-2 | Blank-Gel@HC-EA-4 | MLT-Gel-1 | MLT-Gel@HC-EA-2 |
| MDSC cells | Control-1 | PBS-3 | EA-2 | MLT-1 | Blank-Gel@HC-EA-2 | MLT-Gel-4 | MLT-Gel@HC-EA-4 |

Table S9. The sample numbers in Figure 7c

| Group  Index | Control | PBS | EA | MLT | Blank-Gel@HC-EA | MLT-Gel | MLT-Gel@HC-EA |
| --- | --- | --- | --- | --- | --- | --- | --- |
| AKT | Control-2 | PBS-2 | EA-2 | MLT-2 | Blank-Gel@HC-EA-2 | MLT-Gel-2 | MLT-Gel@HC-EA-2 |
| NF-κB | Control-2 | PBS-2 | EA-2 | MLT-2 | Blank-Gel@HC-EA-2 | MLT-Gel-2 | MLT-Gel@HC-EA-2 |
| IL-1β | Control-2 | PBS-2 | EA-2 | MLT-2 | Blank-Gel@HC-EA-2 | MLT-Gel-2 | MLT-Gel@HC-EA-2 |
